# Prognostic value of high-expression of miR-17-92 cluster in various tumors: evidence from a meta-analysis

**DOI:** 10.1038/s41598-017-08349-4

**Published:** 2017-08-21

**Authors:** Kaiping Zhang, Li Zhang, Meng Zhang, Yin Zhang, Dengxin Fan, Jiabin Jiang, Liqin Ye, Xiang Fang, Xianguo Chen, Song Fan, Min Chao, Chaozhao Liang

**Affiliations:** 1 Department of Urology, Anhui Provincial Children’s Hospital, Hefei, China; 2 Department of Urology, The First Affiliated Hospital of Anhui Medical University (AHMU) and Institute of Urology, AHMU, Hefei, China

## Abstract

The prognostic value of miR-17-92 cluster high-expression in various tumors remains controversial. Therefore, we conducted this meta-analysis by searching literatures in PubMed, Embase, Cochrane Library, China Biology Medicine disc, China National Knowledge Infrastructure to identify eligible studies. Eventually, we analyzed 36 articles that examined 17 tumor types from 4965 patients. Consequently, high-expression of miR-17-92 cluster in various tumors was associated with unfavorable overall survival in both univariate (HR = 2.05, 95%CI: 1.58–2.65, *P*<0.001) and multivariate (HR = 2.14, 95%CI: 1.75–2.61, *P*<0.001) analyses. Likewise, similar results were found in different subgroups of country, test method, miR-17-92 cluster component, sample source and size. Additionally, high-expression of miR-17-92 cluster was linked with poor disease-free survival (Univariate: HR = 1.96, 95%CI: 1.55–2.48, *P*<0.001; Multivariate: HR = 2.18, 95%CI: 1.63–2.91, *P*<0.001), favorable progression-free survival (Univariate: HR = 0.36, 95%CI: 0.16–0.80, *P* = 0.012; Multivariate: HR = 1.55, 95%CI: 0.79–3.05, ﻿*P*﻿ = 0.201) and poor cancer specific survival in univariate rather than multivariate analyses (Univariate: HR = 1.77, 95%CI: 1.21–2.60, *P* = 0.004; Multivariate: HR = 1.77, 95%CI: 0.80–3.92, *P* = 0.160). However, no association of miR-17-92 cluster high-expression was detected with recurrence or relapse-free survival. In summary, this meta-analysis towards high-expression of miR-17-92 cluster has indicated poor prognosis of various cancers. Notably, future studies comprising large cohort size from multicenter are required to confirm our conclusions.

## Introduction

MircoRNAs (miRNAs) are small and single-stranded noncoding RNAs consist of approximately 18–22 evolutionarily conserved nucleotides in length. By binding complementary sequences in the 3′ untranslated region (3′-UTR) of mRNAs, miRNAs either mediate translational suppression or direct mRNAs degradation. Consequently, the mRNAs translation repression or destabilization will lead ﻿to down-regulated expression of the encoded proteins^1, 2^. Meanwhile, as more than half of the sequences encoding miRNAs are located in tumor-associated genomic regions or fragile sites and accumulated evidence has revealed that miRNAs may participate in various cancer-related biological processes including apoptosis, differentiation, proliferation, stress response and metabolism^3–5^, it is widely-accepted that deregulated expression of miRNAs might be used as a novel kind of biomarkers for early cancer diagnosis or prognosis prediction^6^.

The miR-17-92 cluster is a typical and most extensively studied example of miRNAs, which located at chromosomal locus 13q31.3 and encoded the miR-17, miR-18a, miR-19a/b, miR-20a, and miR-92a. Recent studies have reported that miR-17-92 cluster was frequently overexpressed in various cancer types, and played critically suppresive role in degradation or inhibition of its target genes^7–9^. Despite the miR-17-92 cluster has shown great potential in prediction of cancer prognosis, the concretely prognostic value of highly-expressed miR-17-92 cluster in various cancer types remains controversial. However, meta-analysis can explore the authentic and comprehensive results through incorporating all available evidences to get a relatively precise and accurate estimation using statistical software^10^. Thus, we conduct a meta-analysis to assess the possible correlations between high-expression of miR-17-92 cluster with cancer prognosis, which efforts may hold great promise in exploring some novel potential biomarkers for monitoring therapeutic efficacy and prognosis of various cancers.

## Methods

### Ethics statement

The PRISMA statement was used to performed the current meta-analysis^11^. No patient’s privacy or clinical samples were involved in this study, hence the ethical approval was not required.

### Search strategy

Literature resources including PubMed, Cochrane Library, Embase, CBM and CNKI were searched for eligible literatures, using the terms (“microRNA OR miRNA OR miR-17 OR miR-18 OR miR-19 OR miR-20 OR miR-92 OR miR-17-92 cluster”), (“survival OR prognosis OR prognostic”) and (“cancer OR tumor OR tumour OR neoplasm OR neoplasma OR neoplasia OR carcinoma OR cancers OR tumors OR tumours OR neoplasms OR neoplasmas OR neoplasias OR carcinomas”). Last search of current investigation was updated on November 25, 2016. Additionally, the publication language was only limited to English and Chinese. In case of omission, we identified the reference lists of the relevant articles and review articles to seek for the potentially relevant studies. We did not contract the corresponding authors if the relevant data were unavailable.

### Inclusion and exclusion criteria

Studies met the following criteria could be identified: (1) clinical study about the association of high-expression of miR-17-92 cluster with cancer prognostic value; (2) relevant available data of the hazard ratio (HRs) and their corresponding 95% confidence interval (CIs) to evaluate its associations could be obtained; (3) patients prognostic outcomes including overall survival (OS), cancer-specific survival (CSS), relapse-free survival (RFS), progression-free sur-vival (PFS), disease-free survival (DFS). Studies met the following four criteria were excluded: (1) the available data regarding about associations were absent; (2) similar or duplicate study (when the same or similar cohort was applied, after careful examination, the most complete information was included); (3) other types of articles including reviews or abstracts; (4) studies were involved with cells lines or animal models.

### Data extraction

Based on the inclusion and exclusion criteria, we extracted the relevant information from each eligible publication. If disagreements were noticed, we are clearly open to discussion by each other (Kaiping Zhang and Li Zhang), or reviewed by a third author (Min Chao). The data on first author, publication year, study country, age, cancer type, stage range, miRNAs category, sample source, follow-up time, test method, sample size, survival outcome, analysis method, HR and 95%CI and the cut-off value were extracted. We have not contacted any author of the original researches even though the essential information could not be available. Besides, study country came from China and others. Sample source was stratified into tissue, blood, formalin-fixed and paraffin-embedded (FFPE) and tissue microarray (TMA). Test method included immunohistochemistry (IHC), *in situ* hybridization (ISH) and reverse transcription-polymerase chain reaction (RT-PCR); sample sizes were separated into ≥100 and <100 and cancer types included solid cancer and others. Analysis methods were divided into univariate analysis and multivariate analysis.

### Statistical analysis

We explored the association of high-expression of miR-17-92 cluster with cancer prognostic value by applying Review Manager software (RevMan 5, The Cochrane Collaboration, Oxford, UK) and STATA software (Version 12.0, Stata Corpotation, College Station, TX). HR and 95% CI were collected for assessing the prognostic value of highly-expressed miR-17-92 cluster in various cancers. Meanwhile, the heterogeneity has been assessed via chi-square-based Q and I^2^ test across studies (no heterogeneity I^2^<25%, moderate heterogeneity I^2^ = 25–50%, extreme heterogeneity I^2^ > 50%)^12^. In case of extreme heterogeneity (I^2^ > 50% or *P* < 0.01 for Q test), we used random-effects (DerSimonian and Laird method) model^13^. Otherwise, fixed-effects (Mantel-Haenszel method) model was introduced^14^. One-way sensitivity analyses individually removed publications in meta-analysis were conducted to assess results’ stability. It mainly explored the impact of specific study upon mixed HR. The Begg’s funnel plot was performed to evaluated the publication bias. *P* value less than 0.05 indicated that there was a bias of study^15^. Additionally, different subgroups consisted of country, test method, sample source, miR-17-92 component, sample size and cancer type were conducted.

## Results

### Characteristics of the studies

**Figure 1:**
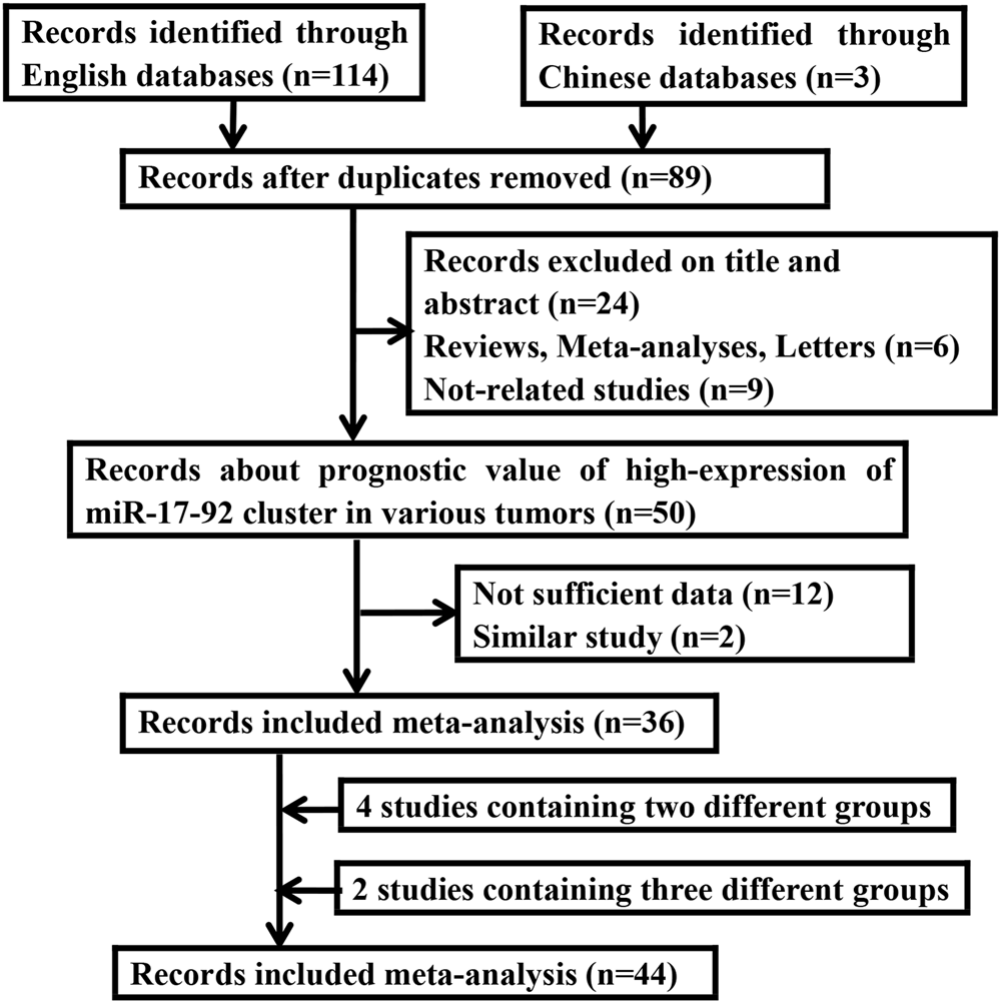
Flow diagram of the study selection process in the meta-analysis.

As a result, 36 studies consisted of 4965 samples satisfied the eligible studies^16–51^ (Fig. 1). The principal characteristics of the included studies were summarized in Table 1. Of these studies, Li’s^18^ study was involved with two different cohorts of Tianjin cohort and Xiangya cohort. Wu *et al*.^27^ designed a multiphase study to identify tissue and serum miRNAs expression, respectively. Fang *et al*.^30^ studied miRNAs expression profiles in colorectal cancer (CRC) patients, comparing chemoresistant and chemosensitive groups. Patients were randomly divided into the training set, internal testing set and independent validation set to search for prognostic value of highly-expressed miR-17-92 cluster in stage II colon cancer by Zhang *et al*.^31^. Ma *et al*.^43^ conducted a study to detect the prognostic value of high-expression of miR-17-5p in CRC using RT-PCR and ISH methods. The expression of miRNAs was measured by RT-PCR in tissues from non small cell lung cancer (NSCLC) patients that originated from Maryland, Norway and Japan by Saito *et al*.^46^. As mentioned above, we enrolled them independently into meta-analysis. Eventually, this meta-analysis was established based on 44 studies (Table 2).

**Table 1:** Main characteristics of the eligible studies.

First author	Year	Country	Age, Median (range)	Cancer type	Stage range	MicroRNA	Sample size	Follow-up, Median (range)	Outcome
Robaina, M.C.^16^	2016	Brazil	7.4 (2–18)	BL	I–IV	miR-17	39	38.5 (1–69)	OS
Ren, C.^17^	2016	China	NA	GC	I–IV	miR-92a	180	85.2 (79.2–97.2)	OS
Li, X.G.^18^	2016	China	43 (13–72)	GBM	I–IV	miR-17	108	NA	OS
Chen, Y.J.^19^	2015	China	NA	GC	I–III	miR-18a	90	NA	OS
Xi, Y.F.^20^	2015	China	18 (3–73)	T-LBL	I–IV	miR-17, miR-19	57	NA (1–156)	OS
Su, X.P.^21^	2015	China	NA	HCC	I–IV	miR-92a	90	NA	OS
Li, J.^22^	2015	China	58.7 (NA)* 56.6 (NA)**	CRC	II–III	miR-17-3p	175	36 (33.0–38.1)* 32 (27.5–35.0)**	DFS
Hao, M.^23^	2015	China	57.5 (33–83)	MM	I–III	miR-19a	108	13.5 (NA)	OS/DFS
Ge, Y.Z.^24^	2015	China	49.5 (42–62)	RCC	I–IV	miR-19a	58	63.4 (31.5–86.1)	RFS
Guo, Y.H.^25^	2015	China	NA	HCC	I–IV	miR-19	51	NA	OS
Xu, X.L.^26^	2014	China	63 (45–81)	ESCC	I–IV	miR-17/miR-18a/miR-19a	105	34.5 (0.89–52.0)	OS/PFS
Wu, C.H.^27^	2014	China	NA	NSCLC	I–III	miR-19b	155	29.0 (23.0–35.0)	OS
Su, Z.X.^28^	2014	China	69.0 (NA)	GC	T1–T4	miR-18a	82	49.8 (NA)	DFS/CSS
Lin, H.M.^29^	2014	Australia	68 (46–87)	PCa	NA	miR-20a	97	12 (3–62)	OS
Fang, L.^30^	2014	China	59 (NA)	CRC	I–IV	miR-17-5p	376	NA	OS
Zhang, J.X.^31^	2013	China	65 (NA)	CC	II	miR-20a-5p	735	66 (50–86)	DFS
Zhou, T.^32^	2013	China	NA	CRC	I–IV	miR-92a	82	NA	OS
Sanfiorenzo, C.^33^	2013	France	65 (NA)	NSCLC	I–III	miR-20a-5p	52	NA	DFS
Mitani, Y.^34^	2013	USA	NA	ACC	I–IV	miR-17/miR-20a/miR-92a	30	NA	OS
Liu, G.H.^35^	2013	China	57.09 (20–89)	CRC	I–IV	miR-92a	166	36.4 (4–53)	OS
Lin, Q.^36^	2013	China	58 (NA)	NSCLC	I–III	miR-19a	201	19 (NA)	OS
He, H.C.^37^	2013	China	59.80 (43- 86)	PCa	T2A–T4	miR-19a	104	NA	RFS
Zheng, J.^38^	2012	China	NA	HCC	I–IV	miR-17-5p	96	NA	OS
Chen, Q.^39^	2012	China	NA	LC	III–IV	miR-17-5p	221	NA	OS
Yu, G.^40^	2012	China	63.0 (35–90)	CC	I–IV	miR-17/miR-18a/miR-19a/miR-19b	48	59.5 (5–66)	OS
Wang, M.^41^	2012	China	NA	GC	I–IV	miR-17-5p/miR-20a	65	36 (NA)	OS
Nilsson, S.^42^	2012	Sweden	65 (NA)	BCa	I–III	miR-92a	117	78 (NA)	RFS
Ma, Y.^43^	2012	China	69 (30–87)	CRC	I–IV	miR-17-5p	425	45.60 (0.20–88.47)^#^ 44.60 (0.17–86.53)^##^	OS
Chen, L.^44^	2012	China	60 (25–74)	HCC	I–IV	miR-17-5p	120	20 (2–46)	OS/DFS
Valladares, A.M.^45^	2011	Spain	62.5 (45–76)	GIC	I–IV	miR-17	33	35 (0–90)	OS/PFS
Saito, M.^46^	2011	USA Norway Japan	63.6 (32–90) 64.4 (37–82) 59.6 (30–76)	NSCLC	I–III	miR-17	89/37/191	NA	CSS/CSS/RFS
Marchini, S.^47^	2011	Italy	52 (21–82)	EOC	I	miR-20a	89	NA	OS/PFS
Liu, R.^48^	2011	China	NA	PC	III–IV	miR-20a	38	NA	OS
Chen, Z.L.^49^	2010	China	60 (43–75)	ESCC	I–III	miR-92a	65	74 (6–102)	OS
Yu, J.^50^	2010	Japan	65.5 (36–86)	PC	I–IV	miR-17-5p	80	NA	OS
Díaz, R.^51^	2008	Spain	69 (NA)	CC	I–IV	miR-17-5p	110	68 (68–99)	OS/DFS

NA, Not available; BL, Burkitt lym phoma; GC, Gastric cancer; T-LBL, T-cell lymphoblastic lymphoma; CRC, Colorectal cancer; RCC, Renal cell carcinoma; ESCC, Esophageal squamous cell carcinoma; MM, Multiple myeloma; NSCLC, Non-small cell lung cancer; LC, Lung cancer; CC, Colon cancer; GBM, Glioblastoma; ACC, Adenoid cystic carcinoma; BCa, Breast cancer; HCC, Hepatocellular carcinoma; GIC, Gastrointestinal cancer; EOC, Epithelial ovarian cancer; PC, Pancreatic cancer; PCa, Prostate cancer; OS, Overall survival; DFS, Disease-free survival; PFS, Progression-free survival; RFS, Recurrence or relapse-free survival; CSS, Cancer specific survival; *Tianjin cohort; **Xiangya cohort; ^**#**^RT–PCR cohort; ^**##**^ISH cohort.

**Table 2:** miR-17-92 cluster evaluation and survival data of the selected studies.

First author	Country	Test method	Cancer type	MicroRNA	Sample source	Outcome	HR (95%CI)	Cut-off value
Robaina, M.C.^16^	Brazil	RT-PCR	BL	miR-17	FFPE	OS	(M)8.945 (2.150–37.212)	Mean
Ren, C.^17^	China	ISH	GC	miR-92a	FFPE	OS	(U)2.94 (2.01–4.31)/(M)3.34 (1.67–6.70)	>inal score of normal paracancerous tissue
Li, X.G.^18^	China	RT-PCR	GBM	miR-17	Tissue	OS	(U)6.2 (1.3–18.6)/(M)5.1 (0.8–15.9)	Mean
Chen, Y.J.^19^	China	IHC	GC	miR-18a	TMA	OS	(U)5.530 (3.169–9.650)/(M)4.615 (2.601–8.188)	The final scores > 3.0
Xi, Y.F.^20^	China	RT-PCR	T-LBL	miR-17/miR-19	FFPE	OS	(M)4.225 (1.249–14.293)/(M)2.179 (1.069–4.440)	Median
Su, X.P.^21^	China	ISH	HCC	miR-92a	FFPE	OS	(U)2.49 (1.37–4.51)	>the average modified histochemical score
Li, J.-1^22^	China	RT-PCR	CRC	miR-17-3p	Blood	DFS	(U)3.72 (1.61–8.60)/(M)3.74 (1.34–10.4)	An optimal cut-off value of 1.613
Li, J.-2^22^	China	RT-PCR	CRC	miR-17-3p	Blood	DFS	(U)3.09 (1.33–7.24)/(M)3.74 (1.34–10.4)	
Hao, M.^23^	China	RT-PCR	MM	miR-19a	Blood	OS/DFS	(M)2.995 (1.167–7.690)/(M)2.787 (1.421–5.468)	Mean
Ge, Y.Z.^24^	China	HiSeq	RCC	miR-19a	Tissue	RFS	(U)9.264 (1.157–74.20)/(M)7.057 (0.636–78.31)	Median
Guo, Y.H.^25^	China	RT-PCR	HCC	miR-19	Tissue	OS	(U)0.180 (0.069–0.471)/(M)0.091 (0.026–0.322)	Median
Xu, X.L.^26^	China	RT-PCR	ESCC	miR-17	Tissue	OS	(M)2.849 (1.258–6.455)	2-ΔΔ^Ct^ > 2 as showing that the target miRNAs was of high expression
				miR-18a		OS/PFS	(M)2.151 (0.990 –4.675)/(M)1.832 (1.044–3.165)	
				miR-19a		OS/PFS	(M)3.471 (1.110–10.857)/(M)3.317 (1.032–10.650)	
Wu, C.H.-1^27^	China	RT-PCR	NSCLC	miR-19b	Tissue	OS	(U)3.591 (1.564–8.246)/(M)3.466 (1.389–8.650)	Median
Wu, C.H.-2^27^	China	RT-PCR	NSCLC	miR-19b	Blood	OS	(U)2.243 (1.328–3.790)/(M)1.800 (1.008–3.216)	
Su, Z.X.^28^	China	RT-PCR	GC	miR-18a	Blood	DFS CSS	(U)1.864 (1.074–3.235)/(M)1.464 (0.776–2.776) (U)1.959 (1.022–3.756)/ (M)1.769 (0.798–3.923)	A cut-off value of 4.85
Lin, H.M.^29^	Australia	RT-PCR	PCa	miR-20a	Blood	OS	(U)1.8 (1.0–3.3)	Median
Fang, L.-1^30^	China	ISH	CRC	miR-17-5p	TMA	OS	(M)1.900 (1.195–3.022)	>score 7
Fang, L.-2^30^	China	ISH	CRC	miR-17-5p	TMA	OS	(M)4.062 (1.235–13.355)	
Zhang, J.X.-1^31^	China	RT-PCR	CC	miR-20a-5p	FFPE	DFS	(U)2.10 (0.97–4.54)	Risk score equals 1
Zhang, J.X.-2^31^	China	RT-PCR	CC	miR-20a-5p	FFPE	DFS	(U)1.69 (0.88–3.26)	
Zhang, J.X.-3^31^	China	RT-PCR	CC	miR-20a-5p	FFPE	DFS	(U)1.85 (1.25–2.73)	
Zhou, T.^32^	China	RT-PCR	CRC	miR-92a	Tissue	OS	(U)2.947 (1.494–5.813)/(M)2.342 (1.072–5.115)	An average increase of 2.04-fold
Sanfiorenzo, C.^33^	France	RT-PCR	NSCLC	miR-20a-5p	Blood	DFS	(M)2.881 (1.009–8.227)	Median
Mitani, Y.^34^	USA	RT-PCR	ACC	miR-17/miR-20a/miR-92a	Tissue	OS	(M)3.65 (1.27–10.5)/(M)3.65 (1.27–10.5)/ (M)3.21 (1.11–9.34)	A cut off of values > 2
Liu, G.H.^35^	China	RT-PCR	CRC	miR-92a	Blood	OS	(U)10.19 (4.05–25.65)/(M)4.36 (1.64–11.57)	Mean
Lin, Q.^36^	China	RT-PCR	NSCLC	miR-19a	Blood	OS	(U)3.042 (2.082–4.444)/(M)1.438 (1.007–2.052)	More than twofold change
He, H.C.^37^	China	ISH	PCa	miR-19a	Tissue	RFS	(U)0.85 (0.35–1.77)	Mean
Zheng, J.^38^	China	RT-PCR	HCC	miR-17-5p	Blood	OS	(U)2.373 (1.293–4.356)/(M)2.192 (1.024–4.691)	Median
Chen, Q.^39^	China	RT-PCR	LC	miR-17-5p	Blood	OS	(U)1.767 (1.039–3.005)	Median
Yu, G.^40^	China	RT-PCR	CC	miR-17/miR-18a/miR-19a/miR-19b	Tissue	OS	(M)2.67 (1.31–6.82)/(M)1.68 (0.33–3.43)/ (M)0.87 (0.71–4.38)/(M)1.52 (1.09–2.11)/ (M)0.76 (1.51–5.37)/(M)1.42 (1.44–4.00)	Median
Wang, M.^41^	China	RT-PCR	GC	miR-17-5p miR-20a	Blood	OS	(U)1.785 (1.110–2.870) (U)1.818 (1.321–2.502)/(M)1.576 (1.102–2.253)	Median
Nilsson, S.^42^	Sweden	ISH	BCa	miR-92a	FFPE	RFS	(U)0.328 (0.138–0.781)/(M)0.375 (0.145–0.972)	Median
Ma, Y.-1^43^	China	RT–PCR	CRC	miR-17-5p	FFPE	OS	(U)1.68 (1.03–2.74)/(M)2.16 (1.20–3.90)	Median (tumour/non-tumour ratio)
Ma, Y.-2^43^	China	ISH	CRC	miR-17-5p	FFPE	OS	(U)2.58 (1.53–4.34)/(M)2.41 (1.40–4.18)	
Chen, L.^44^	China	RT-PCR	HCC	miR-17-5p	Tissue	OS/DFS	(M)4.96 (1.78–13.82)/(M)1.79 (1.14–2.98)	Median
Valladares, A.M.^45^	Spain	RT-PCR	GIC	miR-17	FFPE	OS/PFS	(M)2.62 (1.55–4.49)/(M)2.11 (1.29–3.45)	Mean
Saito, M.-1^46^	USA	RT-PCR	NSCLC	miR-17	Tissue	CSS	(U)2.00 (1.10–3.61)	Median
Saito, M.-2^46^	Norway	RT-PCR	NSCLC	miR-17	Tissue	CSS	(U)1.23 (0.56–2.70)	
Saito, M.-3^46^	Japan	RT-PCR	NSCLC	miR-17	Tissue	RFS	(U)1.37 (0.80–2.37)	
Marchini, S.^47^	Italy	RT-PCR	EOC	miR-20a	Tissue	OS PFS	(U)0.376 (0·141–1·006)/(M) 0.367 (0·115–1·172) (U)0.356 (0.159–0.801)/(M) 0.392 (0.142–1.080)	Median
Liu, R.^48^	China	RT-PCR	PC	miR-20a	Blood	OS	(U)0.56 (0.24–1.34)/(M)0.53 (0.17–1.64)	Risk score > 5.95
Chen, Z.L.^49^	China	RT-PCR	ESCC	miR-92a	Tissue	OS	(U)2.801 (1.348–5.814)/(M)2.198 (1.030–4.673)	The 75th percentiles of 2-ΔΔCt
Yu, J.^50^	Japan	RT-PCR	PC	miR-17-5p	FFPE	OS	(U)1.8 (1.0–3.1)/(M)0.9 (0.4–1.7)	The median expression 5.69
Díaz, R.^51^	Spain	RT-PCR	CC	miR-17-5p	Tissue	OS/DFS	(U)1.06 (0.47–2.39)/(U)1.13 (0.48–2.68)	The median of 4.35

BL, Burkitt lym phoma; GC, Gastric cancer; T-LBL, T-cell lymphoblastic lymphoma; CRC, Colorectal cancer; RCC, Renal cell carcinoma; ESCC, Esophageal squamous cell carcinoma; MM, Multiple myeloma; NSCLC, Non-small cell lung cancer; LC, Lung cancer; CC, Colon cancer; GBM, Glioblastoma; ACC, Adenoid cystic carcinoma; BCa, Breast cancer; HCC, Hepatocellular carcinoma; GIC, Gastrointestinal cancer; EOC, Epithelial ovarian cancer; PC, Pancreatic cancer; PCa, Prostate cancer; IHC, Immunohistochemistry; ISH, *In situ* hybridization; HiSeq, High-throughput sequencing; RT-PCR, Reverse transcription-polymerase chain reaction; FFPE, Formalin-fixed and paraffin-embedded; TMA, Tissue microarray; OS, Overall survival; DFS, Disease-free survival; PFS, Progression-free survival; RFS, Recurrence or relapse-free survival; CSS, Cancer specific survival; U, Univariate analysis; M, Multivariate analysis; HR, Hazard ratio; CI, Confidence interval.

Of 44 studies, 41 were written in English and 3 were published in Chinese. The sample sizes ranged from 30 to 376. The included tumor types were as follows: 1 burkitt lym phoma (BL), 4 gastric cancer (GC), 1 T-cell lymphoblastic lymphoma (T-LBL), 8 CRC, 1 renal cell carcinoma (RCC), 2 esophageal squamous cell carcinoma (ESCC), 1 multiple myeloma (MM), 7 NSCLC, 5 colon cancer (CC), 1 adenoid cystic carcinoma (ACC), 1 breast cancer (BCa), 4 hepatocellular carcinoma (HCC), 1 gastrointestinal cancer (GIC), 1 epithelial ovarian cancer (EOC), 2 pancreatic cancer (PC), 1 glioblastoma (GBM), 1 lung cancer (LC), 2 prostate cancer (PCa). Meanwhile, 7 ISH, 35 RT-PCR, 1 IHC and 1 HiSeq in test methods were applied. According to the sample source, there were 12 formalin-fixed and paraffin-embedded (FFPE); 16 tissue, 13 blood and 3 tissue microarray (TMA). As for the survival outcomes, 44 eligible studies were divided into 51 datasets: 31 for OS, 3 for PFS, 4 for RFS, 10 for DFS and 3 for CSS. However, the cut-off value for the high-expression of miR-17-92 cluster was inconsistent among these included studies (Table 2).

### Meta-analysis of OS

**Figure 2:**
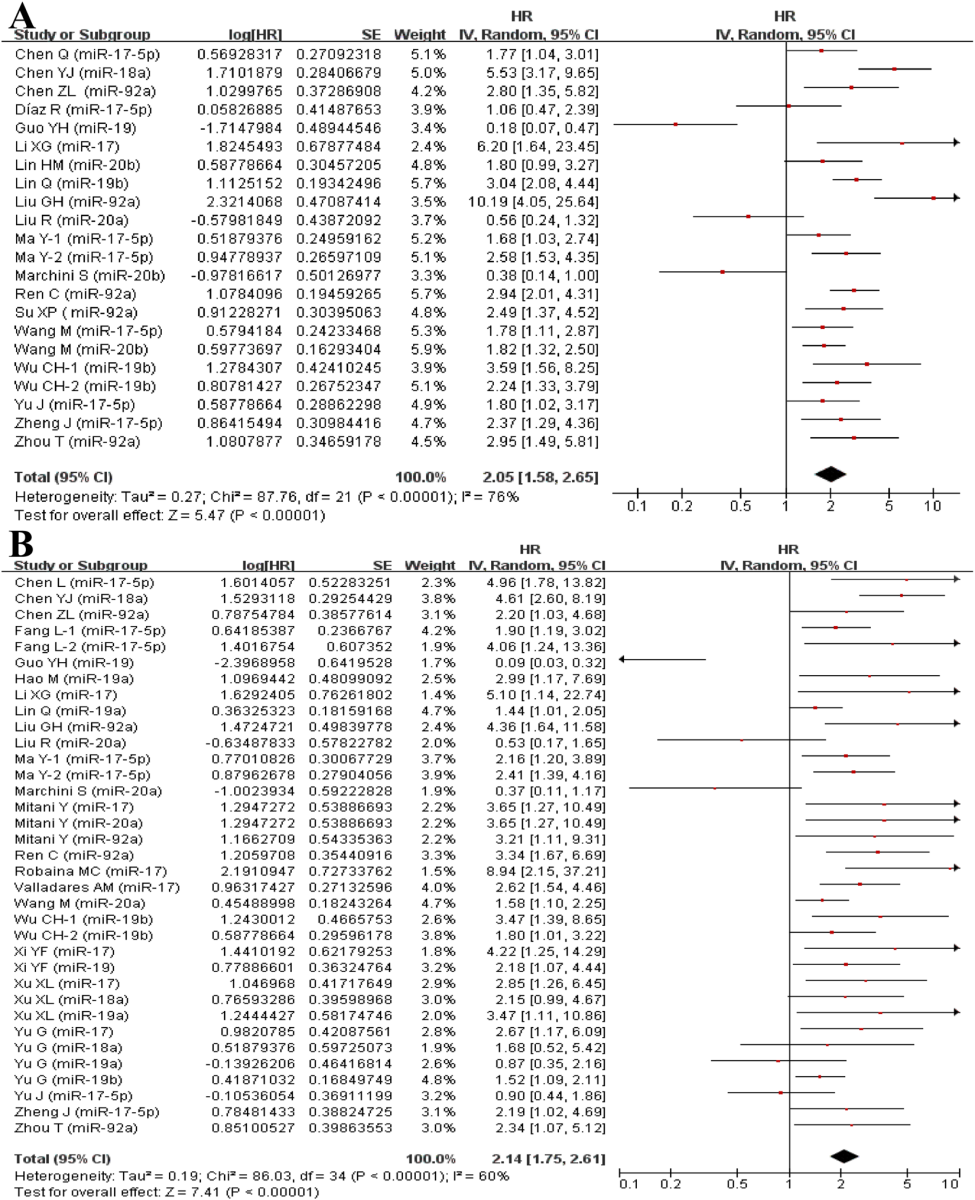
Forest plot of the association between high-expression of miR-17-92 cluster in various tumors and OS under different types of analysis. (**A**) Univariate analysis; (**B**) multivariate analysis). The squares and horizontal lines correspond to the study-specific HR and 95% CI. The area of the squares reflects the weight. The diamond represents the summary HR and 95% CI. CI = confidence interval, HR = hazard ratio.

In univariate analysis, 21 studies were involved in current meta-analysis to assess the prognostic value of highly-expressed miR-17-92 cluster in tumors. High-expression of miR-17-92 cluster in various tumors was associated with unfavorable OS (HR = 2.05, 95%CI: 1.58-2.65, *P* < 0.001) (Fig. 2A). Besides, it seemed that there were certain associations via sub-analyses regarding country, test method, sample source, miR-17-92 component, sample size and cancer type. (Table 3)

**Table 3:** Stratifi ed analysis of the high-expression of miR-17-92 cluster and overall survival.

		No of datasets	HR (95%CI)	*P*-value	I^2^	*P* ^h^	No of datasets	HR (95%CI)	*P*-value	I^2^	*P* ^h^
All		21	**2.05 (1.58–2.65)**	0.000	76.1%	0.000	27	**2.14 (1.75–2.61)**	0.000	60.5%	0.000
Country	China	17	**2.30 (1.75–3.02)**	0.000	75.9%	0.000	22	**2.13 (1.73–2.63)**	0.000	58.2%	0.000
	Others	4	1.18 (0.64–2.16)	0.602	65.6%	0.033	5	**2.18 (1.12–4.25)**	0.021	71.8%	0.002
Test method	RT-PCR	17	**1.83 (1.36–2.46)**	0.000	76.2%	0.000	22	**2.00 (1.60–2.50)**	0.000	60.0%	0.000
	ISH	3	**2.74 (2.08–3.60)**	0.000	0.0%	0.869	4	**2.40 (1.77–3.25)**	0.000	0.0%	0.458
	IHC	1	**5.53 (3.17–9.65)**	0.000	/	/	1	**4.61 (2.60–8.19)**	0.000	/	/
Sample source	Tissue	7	1.44 (0.60–3.43)	0.414	85.8%	0.000	10	**1.98 (1.37–2.88)**	0.000	67.0%	0.000
	Blood	8	**2.11 (1.53–2.90)**	0.000	69.4%	0.001	7	**1.66 (1.35–2.04)**	0.000	42.6%	0.107
	FFPE	5	**2.33 (1.87–2.90)**	0.000	3.4%	0.387	7	**2.35 (1.54–3.00)**	0.000	43.1%	0.091
	TMA	1	**5.53 (3.17–9.65)**	0.000	/	/	3	**3.10 (1.58–6.06)**	0.001	66.5%	0.050
miR-17-92 component	miR-17	8	**1.92 (1.57–2.36)**	0.000	3.5%	0.403	14	**2.41 (1.98–2.94)**	0.000	23.1%	0.204
	miR-18	1	**5.53 (3.17–9.65)**	0.000	/	/	3	**3.19 (2.08–4.90)**	0.000	46.5%	0.154
	miR-19	4	1.55 (0.58–4.11)	0.380	90.1%	0.000	9	**1.54 (1.01–2.36)**	0.044	72.5%	0.000
	miR-20	4	1.02 (0.51–2.04)	0.955	79.1%	0.002	4	1.01 (0.47–2.60)	0.827	73.9%	0.009
	miR-92	5	**3.12 (2.41–4.05)**	0.000	43.4%	0.132	5	**2.89 (2.00–4.18)**	0.000	0.0%	0.797
Sample size	>100	10	**2.60 (1.95–3.47)**	0.000	58.6%	0.010	13	**2.22 (1.87–2.65)**	0.000	16.2%	0.272
	<100	11	**1.61 (1.07–2.41)**	0.022	81.6%	0.000	14	**1.80 (1.31–2.48)**	0.000	71.4%	0.000
Cancer type	Solid cancer	21	**2.05 (1.58–2.65)**	0.000	76.1%	0.000	24	**2.04 (1.65–2.52)**	0.000	62.2%	0.000
	Others	0	/	/	/	/	3	**3.10 (1.91–5.03)**	0.000	9.6%	0.345

RT-PCR, Reverse transcription-polymerase chain reaction; IHC, Immunohistochemistry; ISH, *In situ* hybridization; FFPE, Formalin-fixed and paraffin-embedded; TMA, Tissue microarray; HR, Hazard ratio; CI, Confidence interval; *P* ^h^, *P*-value of heterogeneity test.

In multivariate analysis, 27 studies were included to assess the prognostic value of miR-17-92 cluster. Consequently, high-expression of miR-17-92 cluster in various tumors was associated with unfavorable OS (HR = 2.14, 95%CI: 1.75–2.61, *P* < 0.001) (Fig. 2B). Likewise, a similar result was found in different subgroups (Table 3).

### Meta-analysis of DFS

**Figure 3:**
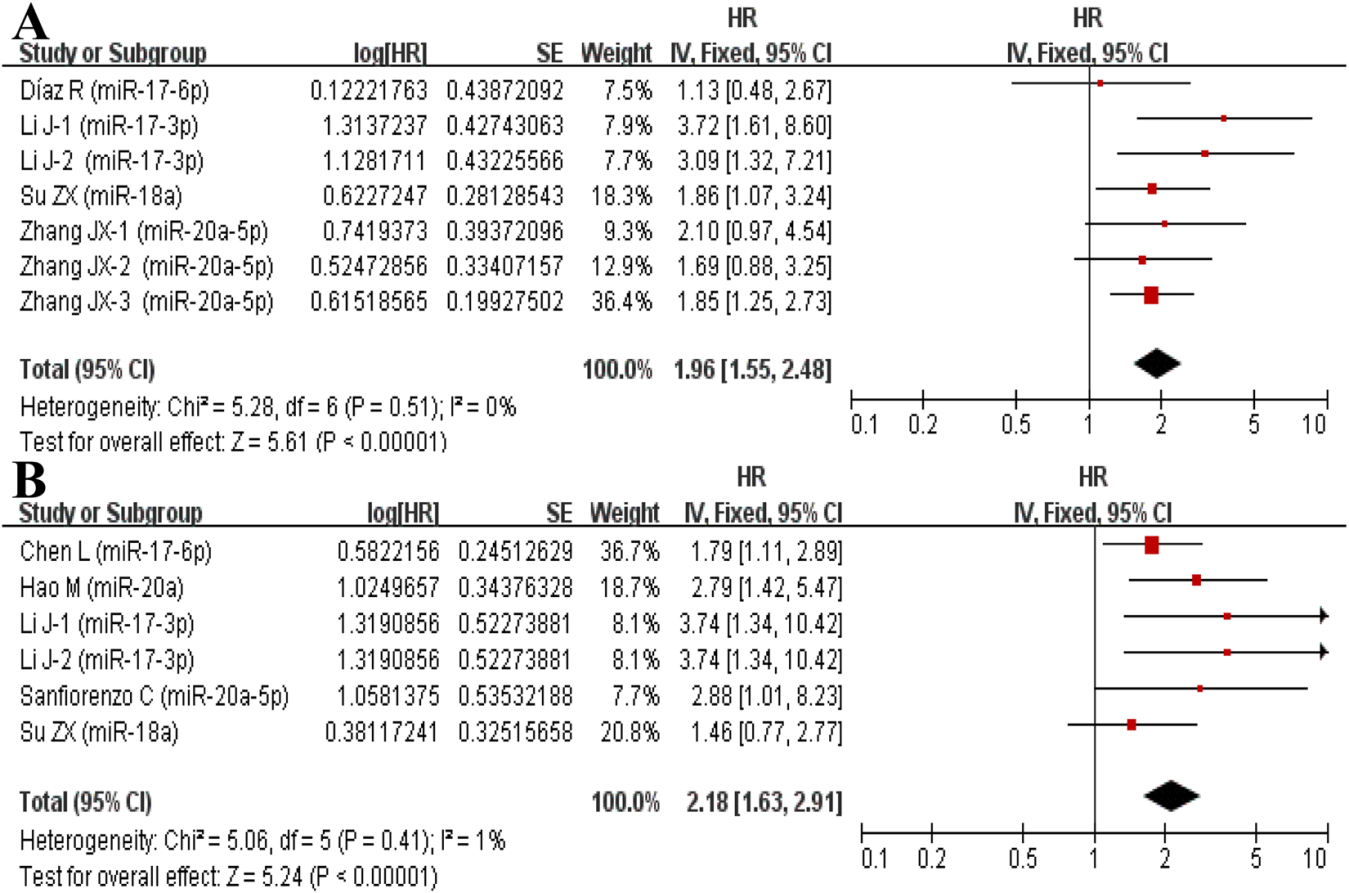
Forest plot of the association between high-expression of miR-17-92 cluster in various tumors and DFS under different types of analysis. (**A**) Univariate analysis; (**B**) multivariate analysis). The squares and horizontal lines correspond to the study-specific HR and 95% CI. The area of the squares reflects the weight. The diamond represents the summary HR and 95% CI. CI = confidence interval, HR = hazard ratio.

7 studies and 6 studies were included in univariate analysis and multivariate analysis, respectively. Ultimately, we found that high-expression of miR-17-92 cluster was linked with poor DFS both in univariate analysis (HR = 1.96, 95%CI: 1.55-2.48, *P* < 0.001) (Fig. 3A) and multivariate analysis (HR = 2.18, 95%CI: 1.63–2.91, *P* < 0.001) (Fig. 3B).

### Meta-analysis of RFS/PFS/CSS

**Figure 4:**
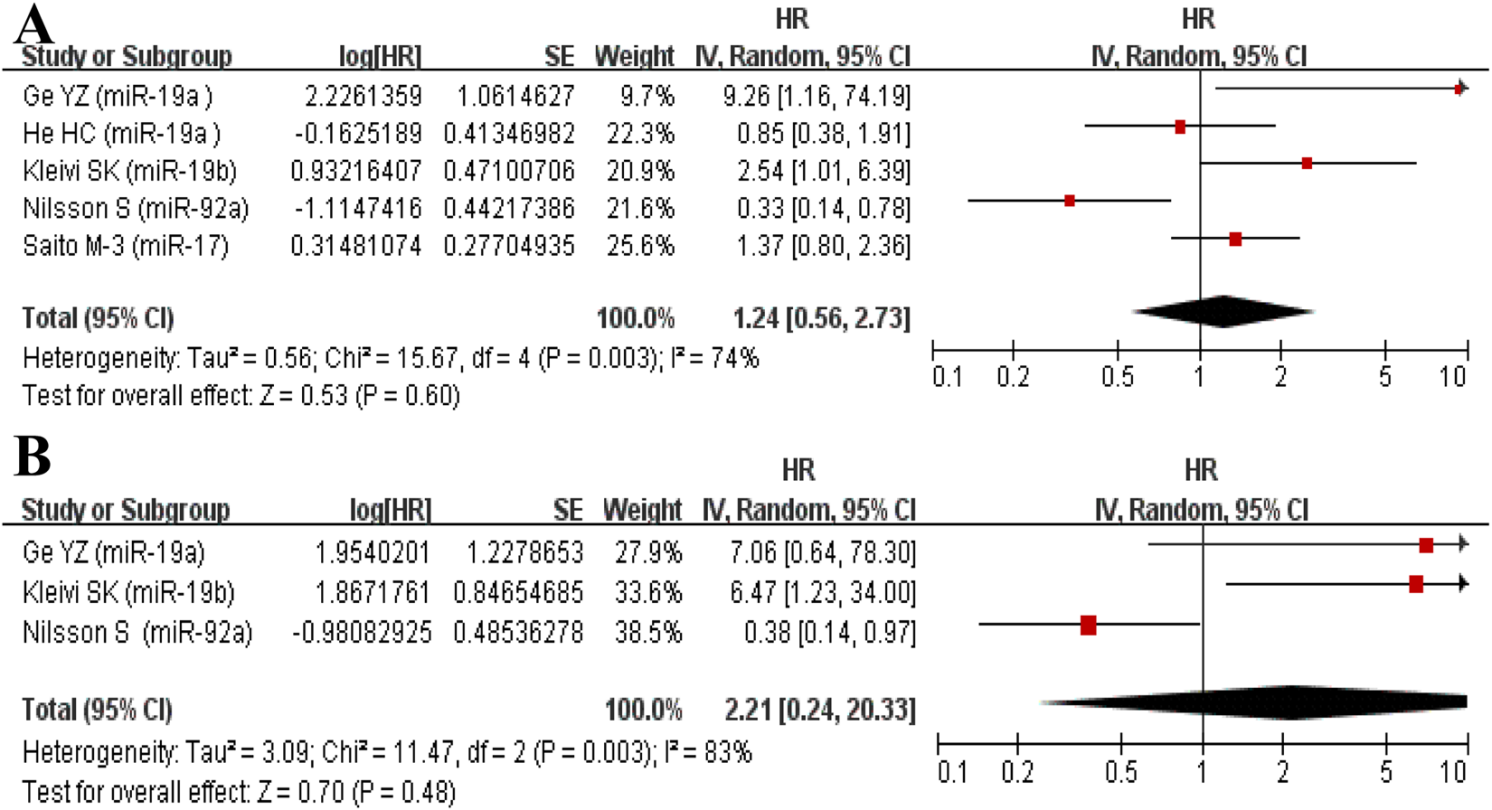
Forest plot of the association between high-expression of miR-17-92 cluster in various tumors and RFS under different types of analysis. (**A**) Univariate analysis; (**B**) multivariate analysis). The squares and horizontal lines correspond to the study-specific HR and 95% CI. The area of the squares reflects the weight. The diamond represents the summary HR and 95% CI. CI = confidence interval, HR = hazard ratio.

In univariate analysis, there were 5studies, 1 study and 3 studies involved with RFS, PFS and CSS, respectively. Correspondingly, 3 studies, 3 studies and 1 study were collected in multivariate analysis. No association of high-expression of miR-17-92 cluster was detected with RFS (Univariate: HR = 1.24, 95%CI: 0.56–2.73, *P* = 0.597; Multivariate: HR = 2.21, 95%CI: 0.24–20.33, *P* = 0.482) (Fig. 4A and B). We also explored that high-expression of miR-17-92 cluster was associated with favorable PFS (Univariate: HR = 0.36, 95%CI: 0.16–0.80, *P* = 0.012; Multivariate: HR = 1.55, 95%CI: 0.79–3.05, *P* = 0.201), and poor CSS in the univariate (HR = 1.77, 95%CI: 1.21–2.60, *P* = 0.004) rather than multivariate analysis (HR = 1.77, 95%CI: 0.80–3.92, *P* = 0.160).

### Sensitivity analysis

**Figure 5:**
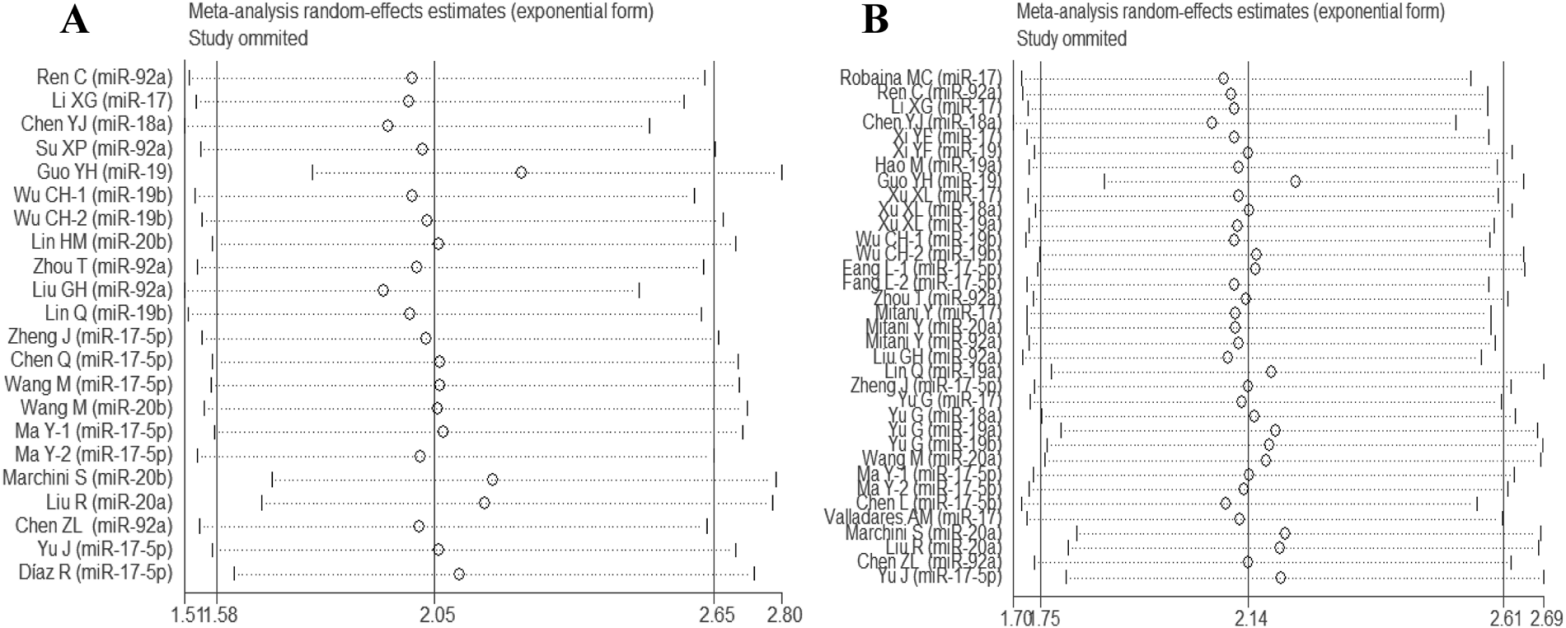
One-way sensitivity analysis of high-expression of miR-17-92 cluster in various tumors with OS under different types of analysis. (**A**) Univariate analysis; (**B**) multivariate analysis). Individually removed the studies and suggested that the results of this meta-analysis were stable.

**Figure 6:**
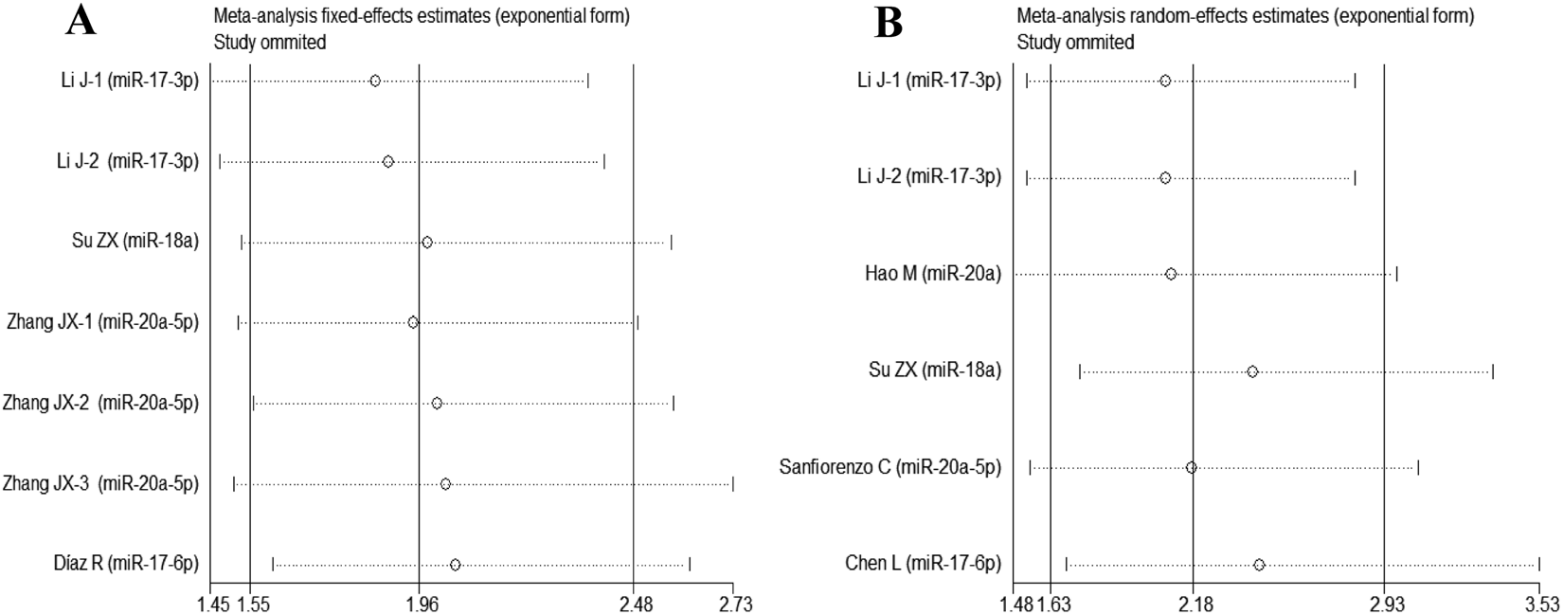
One-way sensitivity analysis of high-expression of miR-17-92 cluster in various tumors with DFS under different types of analysis. (**A**) Univariate analysis; (**B**) multivariate analysis). Individually removed the studies and suggested that the results of this meta-analysis were stable.

**Figure 7:**
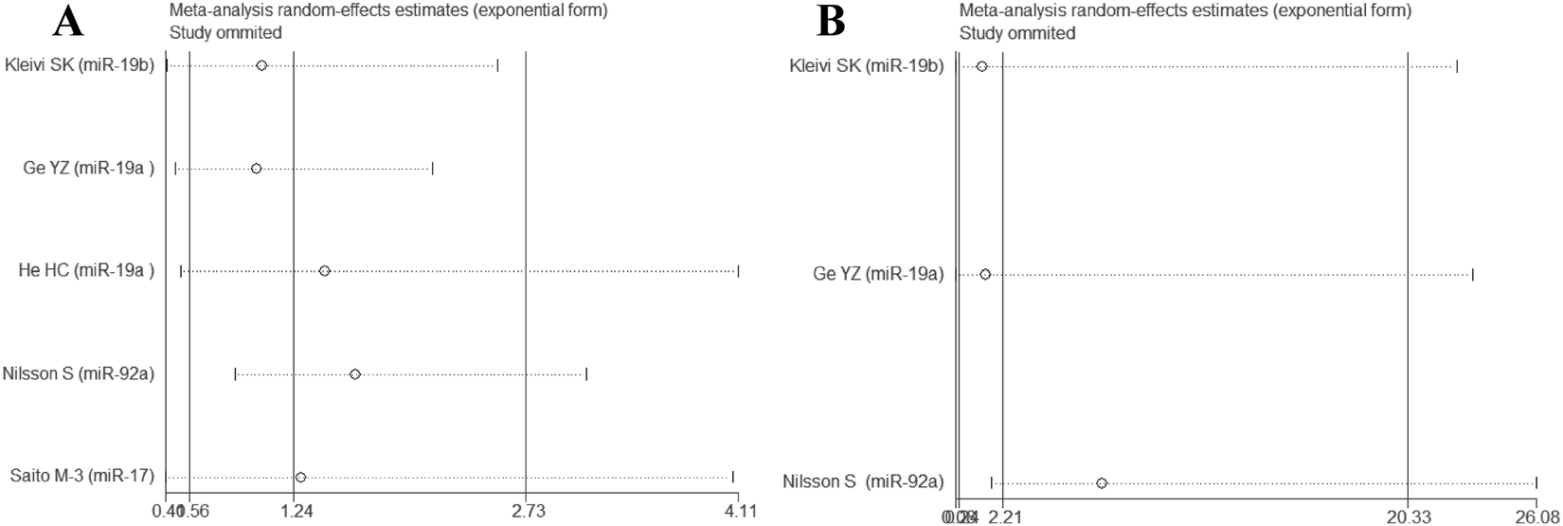
One-way sensitivity analysis of high-expression of miR-17-92 cluster in various tumors with RFS under different types of analysis. (**A**) Univariate analysis; (**B**) multivariate analysis). Individually removed the studies and suggested that the results of this meta-analysis were stable.

Each single study here was deleted at a time to assess the specific effect of the individual data on the pooled HRs, and one-way sensitivity analysis suggested pooled results were relatively stable. Among them, the pooled results of OS, DFS and RFS in both univariate and multivariate analyses were shown in Fig. 5A and B, Fig. 6A and B, Fig. 7A and B, respectively.

### Publication bias evaluation

**Figure 8:**
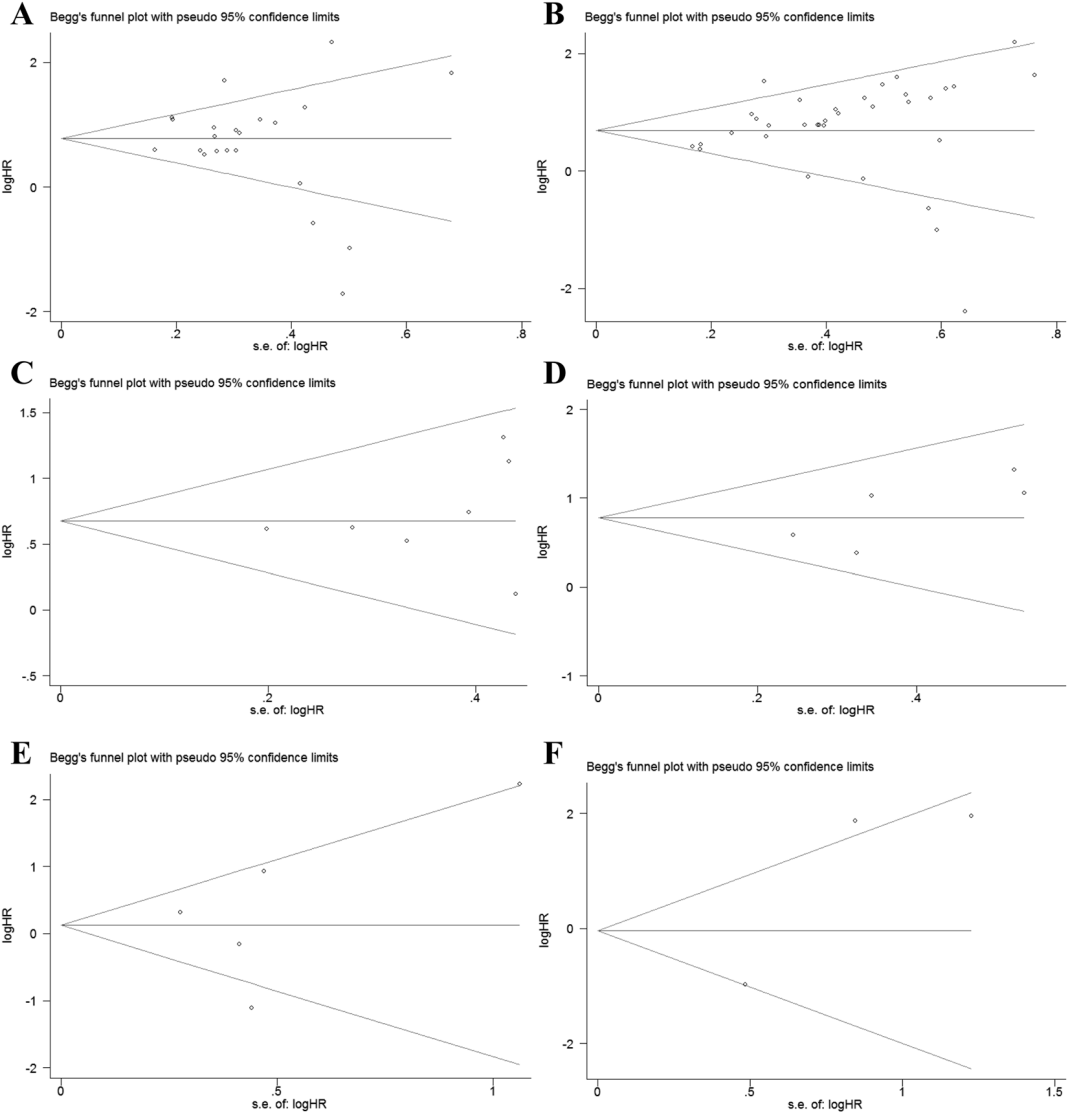
Begg’s funnel plot for publication bias test. (**A**) OS of high-expression of miR-17-92 cluster in various tumors under univariate analysis; (**B**) OS of high-expression of miR-17-92 cluster in various tumors under multivariate analysis; (**C**) DFS of high-expression of miR-17-92 cluster in various tumors under univariate analysis; (**D**) DFS of high-expression of miR-17-92 cluster in various tumors under multivariate analysis; (**E**) RFS of high-expression of miR-17-92 cluster in various tumors under univariate analysis; (**F**) RFS of high-expression of miR-17-92 cluster in various tumors under multivariate analysis;). The x-axis is log (HR), and the y-axis is natural logarithm of HR. The horizontal line in the figure represents the overall estimated log (HR). The two diagonal lines indicate the pseudo 95% confidence limits of the effect estimate. Log (HR) = log-transformed HR, HR = hazard ratio.

In univariate analysis, Begg’s funnel plot indicated that publication bias was not found in meta-analysis of OS (*P* = 0.822 > 0.05, Fig. 8A), DFS (*P* = 0.764 > 0.05, Fig. 8C), RFS (*P* = 0.462 > 0.05, Fig. 8E). Meanwhile, in multivariate analysis, there was no publication bias of OS (*P* = 0.059 > 0.05, Fig. 8B), DFS (*P* = 0.348 > 0.05, Fig. 8D), RFS (*P* = 1.000 > 0.05, Fig. 8F) from Begg’s funnel plot. Moreover, no publication bias was found in each subgroup of mata-analysis of OS. However, we did not evaluated the publication bias for the CSS/PFS meta-analysis because of fewer datasets for meta-analysis.

## Discussion

In recent decades, to explore the clinically useful cancer signatures remains to be the focus of research due to the complexity of cancer. Fortunately, considerable progresses have been achieved to identify the combinatory cancer hallmark-based gene signature sets (CSS sets) for prognostic indicators and therapeutic strategy design. For instance, a model of seven-gene signatures (NHLRC3, ZDHHC21, PRR14L, CCBL1, PTPRB, PNPO and PPIP5K2) was applied to predict OS by dividing colorectal cancer (CRC) patients into low-risk and high-risk groups. Consequently, the poorer OS was detected in high-risk group compared with low-risk CRC patients^52^. Additionally, the benefit from adjuvant chemotherapy for patients with stage II CRC after surgery remains a matter of debate^53^. Gao *et al*. analyzed data from approximately 1000 patients with stage II CRC from 13 independent cohorts and explored eight CSS sets for determining prognosis of patients. The CSS sets accurately stratified patients into low-, intermediate-, high-risk groups, and predicted five-year RFS rates were 94%, 78% and 45%, respectively for 60%, 28% and 12% of patients with stage II disease. Meanwhile, they have addressed that CSS sets-defined high-risk patients with stage II CRC could gain survival benefit from fluorouracil-based adjuvant chemotherapy^54^.

Apart from CSS sets, miRNAs have also added some novel insights into cancer diagnosis and therapy. Meanwhile, as miRNAs mainly regulated gene expression by targeting the 3′-UTR of mRNAs, the post-transcriptional effects could consequently make the target genes potential cancer hallmarks in clinical trials. For instance, the miR-17-92 cluster consisted of miR-17, miR-18a, miR-19a/b, miR-20a, and miR-92a, has been proven to play significantly regulatory roles in development, progression and prognosis of several cancer types. It was previously reported that miR-18-mediated low-expression of target gene TGF-β intimately contributed to prolonged survival of glioblastoma multiforme (GBM) patients^55^. Similarly, miR-19 could exhibit modulatory effect on GSK-3β/β-catenin axis, down-regulated target gene GSK-3β could promote the metastatic potential of lung cancer cells, revealing a poor survival outcome for cancer patients^56, 57^. Besides, miR-20a was able to suppress the hepatocellular cancer cell proliferation and migration by directly targeted RUNX3, while clinical evidences have proved that RUNX3 was negatively associated with tumor progression, lymph node metastasis and poor prognosis^58, 59^. Collectively, the gene targets of miR-17-92 cluster could potentially serve as different cancer hallmarks. These cancer hallmarks constituted an organizing principle that provided a logical framework for understanding the remarkable diversities of neoplastic diseases^60^.

Although it appears reasonable to identify the clinically prognostic value of the dysregulated expression of miR-17-92 cluster itself in various cancers, the investigations focused on the clinical correlation between high-expression of miR-17-92 cluster with cancer prognosis were relatively rare and inconclusive. Moreover, small sample-sized studies lacking statistical power often have resulted in apparently contradicting conclusions. Meta-analysis is a useful tool for providing convincing evidence as it could present inconsistent results from different investigations to get a relatively precise estimation. As far as we know, the current meta-analysis is the first try to comprehensively assess the correlation of high-expression of miR-17-92 cluster with cancer prognosis. Meanwhile, the potential associations were explored in different subgroups. Consequently, the finding of significant correlation between high-expression of miR-17-92 cluster and unfavorable OS/DFS/CSS in various tumors by two different statistical methods is of particular interest. Likewise, similar results were found in different subgroups. However, no association of high-expression of miR-17-92 cluster was detected with RFS. Additionally, we demonstrated that high-expression of miR-17-92 cluster was associated with favorable PFS by two different statistical methods. Probably due to relatively fewer studies of RFS/PFS/CSS, these results remain inconclusive and require further investigation.

Due to significant heterogeneity of the current meta-analysis, careful interpretation and search for influencing factors were required. Firstly, impact of ethnicity on prognosis in patients was considerable, which should be take into consideration when evaluating the prognosis of cancer for patients^61^. It is a well-established fact that formalin-fixation and/or prolonged storage can elicit damage to nucleic acids, further conferring considerable limitation on results^62, 63^. Accordingly, differences in the detection and quantification methods, types and numbers of miRNAs evaluated and sample source should be also considered as potential sources of heterogeneity. We performed further subgroup analyses according to country, test method, sample source, miR-17-92 cluster component and sample size. All of the subgroup analyses also indicated that high-expression of miR-17-92 cluster was associated with poor OS. As for PFS/RFS/DFS/CSS, we did not perform subgroup analyses due to relatively fewer eligible studies.

Actually, our meta-analysis has its limitations. Firstly, only published studies might not provide sufficient evidences. Additionally, studies regarding various tumors without a consistent cut-off value may influence the ultimate results and two eligible studies^20, 25^ did not clearly illustrate the hypotype of miR-19. Meanwhile, the heterogeneity suggested that potential or undiscovered factors including adjustment for surgery, radiation, chemotherapy, socioeconomic status, tumor characteristics, and so on might be ignored. Whereas, in spite of aforementioned limitations, a certain relationship of high-expression of miR-17-92 in prognostic value was found in current meta-analysis.

In conclusion, the current study is the first original meta-analysis to address the correlation between the miR-17-92 cluster expression and prognostic value for patients. A marginally significant association was explored in overall population as well as the corresponding subgroups. Concretely, it presented that high-expression of miR-17-92 cluster might be associated with poor OS/DFS/CSS and favorable CSS to some extent, while no association was detected between high miR-17-92 expression with RFS. Notably, due to relatively fewer studies of RFS/PFS/CSS, these results still require further verification in the near future.
